# Clinical progress note: Varicella Zoster

**DOI:** 10.1002/jhm.70126

**Published:** 2025-08-13

**Authors:** Sirey Zhang, Adam L. Hersh, T. W. Jones

**Affiliations:** ^1^ Division of Infectious Diseases University of Utah Pediatrics Salt Lake City Utah USA

## Abstract

Varicella Zoster virus (VZV) is the etiologic agent responsible for varicella and herpes zoster (shingles). Nonimmune children and adults acutely infected with VZV typically experience a vesicular and pruritic rash that progresses from the face and trunk and generalizes to the extremities, accompanied by an oral enanthem along with symptoms of fever and malaise. Later, the virus may reactivate from dormancy in the dorsal root ganglia, leading to a stereotypical, unilateral, painful, vesicular rash limited to one or two dermatomes. While either pattern of infection is usually self‐limited in healthy children, more severe complications including death may occur among immunocompromised, pregnant, or adult patients. VZV vaccines have greatly reduced morbidity and mortality since their introduction more than 30 years ago. Hospitalists should be prepared to recognize and treat patients with VZV infection, particularly in an era of increasing vaccine hesitancy.

## INTRODUCTION

Varicella Zoster virus (VZV) is a highly contagious pathogen in the herpesvirus category that presents in two distinct clinical forms: varicella (“chickenpox”) and herpes zoster (“shingles”). VZV is primarily spread through direct contact with fluid from the vesicles of an infected person or through airborne droplets released by coughing or sneezing, manifesting after an incubation period of 10–21 days.^1^

In younger children, the disease presents as a mild illness characterized by an itchy rash and fever. However, in adolescents, adults, and immunocompromised individuals, VZV can lead to severe complications, including pneumonia and encephalitis. After an acute infection, the virus can remain latent in the nervous system and later reactivate as zoster, a painful manifestation that is transmissible through direct contact with its characteristic lesions, and sometimes distinguished by residual neuropathic pain even after resolution of cutaneous manifestations.^2^ The introduction of the varicella and zoster vaccines has significantly reduced the incidence of both chickenpox and shingles. Nonetheless, outbreaks still occur, particularly in populations with low vaccination coverage. This clinical progress note reviews the vaccination impact, clinical course, management, and complications of VZV.

## SEARCH STRATEGY

A PubMed search was conducted using search criteria including Varicella Zoster Virus, Varicella, and Shingles, focusing on vaccines, diagnostics, and manifestations to review the most recent literature.

## VARICELLA VACCINES AND RECOMMENDED SCHEDULE

The live varicella vaccine was first developed by Michiaki Takahashi in 1974 in Japan.^3^ Initially, many questioned the utility of a vaccine for a viral illness perceived to be little more than a routine rite of childhood. Second, many feared that as an attenuated virus, it could lead to viral dormancy and later reactivation into herpes zoster—whether this would occur at higher rates compared to natural infection was unknown. However, as increasing numbers of infants with primary immunodeficiency survived into childhood and chemotherapeutic regimens and immunomodulation saved the lives of many more children with malignancies or rheumatologic disorders, the necessity of population‐level protection against varicella became increasingly evident. Furthermore, the vaccine was demonstrated to be quite effective at averting complications of natural varicella among immunocompetent children and adults, such as secondary bacterial pneumonia or cellulitis, as well as more common problems with natural varicella infection, like school absence. It also served as an important protective agent for nonimmune pregnant people and unborn infants. After its utility and safety was demonstrated overseas, it was first introduced to the United States in 1995.^4^

Currently, the varicella vaccine is routinely given in two doses to children. The first dose is given at age 12–18 months, followed by a second dose at age 4–6 years.^5^ In the latter age group, it is either provided on its own (VZV vaccine) or in combination with the Measles, Mumps, Rubella, and Varicella vaccine (MMRV), both of which may have local injection site reactions, such as a rash. The MMRV combined vaccine has a small excess risk of febrile seizure, which is why it is not given to toddlers.

The recombinant zoster vaccine (Shingrix), administered as two intramuscular doses 2 to 6 months apart, is recommended for adults aged 50 years and older, regardless of prior history of shingles or previous receipt of the live zoster vaccine (Zostavax). It demonstrates greater than 90% efficacy in preventing herpes zoster and postherpetic neuralgia for at least 4 years postvaccination, but it does not impact the incidence of primary varicella (chickenpox).^2^ It is known to have mild‐to‐moderate local and systemic reactions, such as fever and malaise.

## VACCINE IMPACT

Before the introduction of the vaccine, natural varicella infection caused approximately 100 deaths each year in the United States, with over 10,000 hospitalizations.^6^, ^7^ Immunocompromised children and adults were at increased risk of morbidity and mortality due to natural varicella infection. Moreover, natural varicella infection among immunocompetent children led to lost household earnings or days in school, as sick children required parental care and isolation at home.^8^ Since the rollout of the vaccine, overall deaths and hospitalizations have been reduced by over 90% in the United States compared to pre‐vaccine levels.^9^ In addition, from 1996 to 2020, the US varicella vaccination program has been estimated to save 118,000 life‐years and generate a net societal savings of $23.4 billion.^8^

The VZV vaccine not only provides protection against natural varicella infection, but it has also been demonstrated to be protective against later reactivation of herpes zoster.^4^ A single dose of varicella vaccine is estimated to be 82% effective at preventing clinical varicella and 98% effective at preventing serious disease.^5^ Two doses of varicella vaccine provide a 92‐95% effectiveness against any clinical varicella disease.

## CLINICAL MANIFESTATIONS AND COMPLICATIONS

After an incubation period of about 2 weeks, varicella presents with a generalized, pruritic, maculopapular and vesicular rash, typically consisting of 250 to 500 lesions on an erythematous base, which usually begins on the face or trunk before spreading; this may be accompanied by headache, myalgia, and fever.^8^ Described as ‘dewdrops on a petal,' these lesions can present in different stages of evolution, differentiating it from Herpes simplex virus. Partially or fully vaccinated patients may occasionally experience breakthrough disease, which is characterized by very few vesicular lesions or a maculopapular rash alone.

During varicella disease, patients are contagious via aerosolized droplet nuclei. Airborne precautions are required to prevent the spread of infection to other nonimmune hosts.

Following an initial infection, the virus becomes latent in the dorsal root ganglia and can later reactivate as herpes zoster (shingles). This latter form may also be contagious, albeit only through direct contact, and presents with a self‐limited but painful vesicular rash in a dermatomal distribution.^10^ This rash usually self‐resolves within several days. A small subset of individuals with shingles may experience ongoing severe pain following resolution of the vesicular lesions, known as post‐herpetic neuralgia.

Immunocompromised children are at higher risk for severe varicella and disseminated zoster. This is especially true for those with T‐lymphocyte depletion, such as children with uncontrolled human immunodeficiency virus (HIV), or hematopoietic stem cell transplants. Other high‐risk groups are those on immunosuppressive therapy; previously healthy children on high‐dose corticosteroids over 2 mg/kg/day of prednisone or equivalent have been reported to develop severe and sometimes fatal varicella.^5^

Complications to natural varicella infection among both immunocompetent and immunocompromised individuals can involve bacterial superinfection like cellulitis, pneumonia, and sepsis, as well as central nervous system disease (acute cerebellar ataxia and encephalitis), thrombocytopenia, and rare issues like glomerulonephritis or hepatitis.^5^ Reye's syndrome (hepatic encephalopathy) has been reported in the context of salicylate use during natural varicella infection; thus, aspirin or bismuth salicylate use is discouraged during both natural infection and within 6 weeks of immunization.

Fetal infection of VZV during the first or early second trimester can lead to fetal death or congenital varicella syndrome, comprising of limb hypoplasia, cutaneous scarring, eye abnormalities, and damage to the central nervous system.^5^ When varicella is placentally transferred within 1 week of delivery, higher rates of newborn mortality are observed as it is believed there is less time for maternal immunoglobulin development, transfer, and protection.^10^

## DIAGNOSIS

Acute varicella infection is generally diagnosed clinically.^10^ When confirmatory testing is needed, polymerase chain reaction (PCR) for viral genetic material may be performed on vesicular fluid or scabs, cerebrospinal fluid (CSF), bronchoalveolar lavage, blood, or amniotic fluid. Vesicular fluid and lesion scabs are considered the diagnostic samples of choice and have the highest yield in PCR testing.^5^ Direct fluorescent antibody testing may also be performed from scrapings of vesicle bases early in illness. Serologic testing (IgG only) can be used to confirm immunity from prior immunization or infection, but this can be negative in those who did receive two vaccines, making it an insensitive confirmation method—prior immunity is best confirmed by documented vaccine receipt or a phyisican‐diagnosed past episode of clinical varicella.^11^ IgM antibody testing is not felt to be useful for diagnosing recent acute infection due to high rates of false‐positive and false‐negative results.^5^

## TREATMENT

For children presenting within a healthcare facility or clinic with suspected varicella infection, airborne precautions should be implemented, and the facility's infection prevention team should be contacted immediately.

Among immunocompetent children under age 12, management of natural varicella infection is largely focused on supportive care.^5^ Secondary bacterial complications like pneumonia or cellulitis may require antibiotic therapy.

Antiviral therapy with acyclovir or valacyclovir is recommended for those with natural varicella infection at higher risk of complications, such as individuals over age 12, people with chronic skin or pulmonary disease, children on long‐term salicylate therapy, or those with immunocompromised status.^5^

When a previously healthy, nonimmune individual is exposed to an active case of varicella, postexposure prophylaxis should be undertaken by giving the varicella vaccine within 3‐5 days after exposure.^5^ This may prevent or favorably modify the course of natural disease.

Varicella Zoster Immune Globulin (VZIG) postexposure prophylaxis should be administered for nonimmune high‐risk individuals, like immunocompromised patients, pregnant people, and newborns, who have had significant exposure to a case of varicella. Example exposures that are considered significant include individuals residing in the same household, playmates who have had indoor face‐to‐face playtime for 5 min or more, patients in the same 2‐ to 4‐bed room or adjacent beds in a ward, or any face‐to‐face contact with an infectious staff member, visitor, or patient.^5^ Newborns born to pregnant people who develop varicella within a window of 5 days preceding delivery or 2 days following delivery should also receive VZIG.^5^ Infectious disease specialists and infection preventionists should be consulted in all such suspected exposure cases. VZIG should be given within 10 days of exposure, with live vaccines delayed for 5 months afterward.

Antiviral chemoprophylaxis with acyclovir or valacyclovir is recommended in select cases, such as immunocompromised patients or unvaccinated high‐risk contacts who cannot receive VZIG within the 10‐day window mentioned above (because of contraindications or lack of availability).^5^ Such antiviral chemoprophylaxis should be initiated within 7 days postexposure and continued for 7 days.

## CONCLUSION

VZV has two distinct clinical presentations, namely the acute presentation of varicella and the subsequent reactivation of zoster. While a primary infection is typically benign and self‐limiting in otherwise healthy individuals, the infection poses significant morbidity and mortality to adolescents and adults, pregnant people, individuals with malignancies or immunocompromised status, and developing fetuses in the first trimester or just before delivery. The VZV vaccine, now in use for almost 30 years, has significantly reduced the number of hospitalizations and deaths from complications. The shingles vaccine has led to a substantial reduction in the incidence of herpes zoster and its complications, including postherpetic neuralgia, among older adults.

## CONFLICT OF INTEREST STATEMENT

The authors declare no conflicts of interest.
